# Combinational Inhibitory Action of *Hedychium spicatum* L. Essential Oil and γ-Radiation on Growth Rate and Mycotoxins Content of *Fusarium graminearum* in Maize: Response Surface Methodology

**DOI:** 10.3389/fmicb.2018.01511

**Published:** 2018-07-31

**Authors:** Naveen K. Kalagatur, Jalarama R. Kamasani, Chandranayaka Siddaiah, Vijai K. Gupta, Kadirvelu Krishna, Venkataramana Mudili

**Affiliations:** ^1^Food Microbiology Division, Defence Food Research Laboratory, Mysuru, India; ^2^Freeze Drying and Processing Technology Division, Defence Food Research Laboratory, Mysuru, India; ^3^Department of Biotechnology, University of Mysore, Mysuru, India; ^4^Department of Chemistry and Biotechnology, Tallinn University of Technology, Tallinn, Estonia; ^5^DRDO-BU-Centre for Life Sciences, Coimbatore, India

**Keywords:** mycotoxins, *Fusarium graminearum*, *Hedychium spicatum*, deoxynivalenol, zearalenone, essential oil, γ-radiation, response surface methodology

## Abstract

Nowadays, contamination of agricultural commodities with fungi and their mycotoxins is one of the most annoying with regard to food safety and pose serious health risk. Therefore, there is a requisite to propose suitable mitigation strategies to reduce the contamination of fungi and mycotoxins in agricultural commodities. In the present study, combinational inhibitory effect of *Hedychium spicatum* L. essential oil (HSEO) and radiation was established on growth rate, production of deoxynivalenol (DON) and zearalenone (ZEA) by *Fusarium graminearum* in maize grains. The HSEO was obtained from rhizomes by hydrodistillation technique and chemical composition was revealed by GC-MS analysis. A total of 48 compounds were identified and major compounds were 1,8-cineole (23.15%), linalool (12.82%), and β-pinene (10.06%). The discrete treatments of HSEO and radiation were effective in reducing the fungal growth rate and mycotoxins content, and the complete reduction was noticed at 3.15 mg/g of HSEO and 6 kGy of radiation. Response surface methodology (RSM) was applied to evaluate the combinational inhibitory effect of HSEO and radiation treatments on fungal growth rate and mycotoxins content. A total of 13 experiments were designed with distinct doses of HSEO and radiation by central composite design (CCD) of Stat-Ease Design-Expert software. In combinational approach, complete reductions of fungal growth, DON, and ZEA content were noticed at 1.89 mg/g of HSEO and 4.12 kGy of radiation treatments. The optimized design concluded that combinational treatments of HSEO and radiation were much more effective in reducing the fungal growth and mycotoxins content compared to their discrete treatments (*p* < 0.05). Responses of the design were assessed by second-order polynomial regression analysis and found that quadratic model was well fitted. The optimized design has larger *F*-value and adequate precision, smaller *p*-value, decent regression coefficients (*R*^*2*^) and found statistically significant (*p* < 0.05). In addition, correlation matrix, normal plot residuals, Box-Cox, and actual vs. predicted plots were endorsed that optimized design was accurate and appropriate. The proposed combinational decontamination technique could be highly applicable in agriculture and food industry to safeguard the food and feed products from fungi and mycotoxins.

## Introduction

Mycotoxins are toxic secondary metabolites produced by filamentous fungi on agricultural products, which cause acute or chronic toxic effects in farm animals and humans called mycotoxicosis (Schirone et al., 2016; Du et al., 2017). The contamination of agricultural products with fungi occurs during pre-and post-harvesting stages due to inappropriate and unhygienic practices (Bernhoft et al., 2012). The Food and Agricultural Organization (FAO) of the United Nations estimate that ~25% of agricultural products are contaminated with fungi and mycotoxins worldwide (Pitt and Hockings, 2009). The fungal infestation brings intolerable alterations in appearance, color, texture, flavor, and nutrition of food (Pitt and Hockings, 2009). Therefore, the incidence of fungi and mycotoxins contamination in agricultural products have become one of the foremost issues of farmers, food industry, and government concerning the food safety (Annunziata et al., 2017; Udomkun et al., 2017).

Among the mycotoxigenic fungi, *Fusarium graminearum* has received a wide attention because of its ability to produce a variety of mycotoxins, such as deoxynivalenol (DON), nivalenol (NIV), and zearalenone (ZEA) under diverse climate conditions (Pasquali et al., 2016). Several studies have addressed the toxic effects of DON and ZEA in *in-vitro* and *in-vivo* experiments and documented their genotoxicity, hepatotoxicity, neurotoxicity, immunotoxicity, nephrotoxicity, reproductive, and developmental toxicity, carcinogenicity, etc. (Zinedine et al., 2007; Venkataramana et al., 2014; Schumann et al., 2016; Kalagatur et al., 2017; Gonçalves et al., 2018; Muthulakshmi et al., 2018; Reddy et al., 2018). Moreover, International Agency for Research on Cancer (IARC) has studied the carcinogenic property of DON and ZEA in laboratory animals and classified as the group 3 carcinogens (IARC, 1999).

The DON and ZEA have been detected in wide range of agricultural commodities, such as barley, corn, corn silage, hay, oats, rice, sesame seed, sorghum, and wheat (CAST, 2003; Zinedine et al., 2007). Particularly, Fusarium head blight (FHB) in wheat and barley, and Gibberella ear rot in maize caused by *F. graminearum* is a devastating plant disease of temperate regions and results in yield loss and mycotoxins contamination (Wilson et al., 2018). Most lately, Xu et al. (2018) from North China Plain have detected as high as 95.7% of maize germ contamination with DON and measured average attendance of DON in processed products of maize germs as 163.7–1175.2 μg/kg. In Hungary, Tima et al. (2018) have surveyed the occurrence of DON in maize, wheat and its by-products during the period 2008–2015 and noticed overall mean of 2,159 ± 2,818 μg/kg, which was annoyingly much higher than maximum allowed limit. Besides, Mallmann et al. (2017) have summarized the occurrence of DON and ZEA in barley and wheat grains of Southern Brazil during the middle of 2008–2015 and noticed 67% of DON and 41% of ZEA contamination. Similarly, Tralamazza et al. (2016) have observed 99% of DON and 84% of ZEA contamination in wheat samples originated from Brazil. Also, Ji et al. (2014) have detected 74.4% of wheat contaminated with DON in the FHB epidemic region of Jiangsu province, China. In our previous study (Mudili et al., 2014) 72–94 μg/kg of DON in freshly harvested maize grains from Andhra Pradesh, Karnataka, and Tamil Nadu states of Southern India was noticed. Furthermore, Mishra et al. (2013) have also measured the exposure and risk assessment of DON in the Indian individuals, and DON was identified in 30% of cereal samples and in that 7% of samples were surpassed the FSSR (Food Safety and Standard Regulation, India) limit of 1 mg/kg. In this context, *F. graminearum* and its toxins, DON, and ZEA have a great threat to the agricultural and food industry, especially in warm, and humid climate country like India (Ramana et al., 2011; Mishra et al., 2013; Divakara et al., 2014; Mudili et al., 2014; Aiyaz et al., 2016). In this scenario, there is a need to develop safe strategies to combat the problems of mycotoxigenic *F. graminearum* as well as DON and ZEA contamination in agricultural products.

Since last decade, agriculture and food industry have applied various decontamination practices to safeguard the agricultural products from fungi and mycotoxins (Zinedine et al., 2007; Kanapitsas et al., 2016; Kalagatur et al., 2018a,b). Unfortunately, some methods were undesirable, and only few of them were acceptable by WHO, FAO, EU, JECFA, and other regulatory agencies with some constraints. The application of synthetic fungicidal agents could drive the drug-resistant fungi, environmental pollution, health risk in animals and humans, and its use in food has been not passable and restricted worldwide (Kretschmer et al., 2009). Henceforth, there is a huge demand for bio-fungicides as an alternative to synthetic fungicidal agents (Akocak et al., 2015; George et al., 2016; Iram et al., 2016; Kumar et al., 2016; Sellamani et al., 2016; Kalagatur et al., 2017; Muniyandi et al., 2017). On the other hand, chemical decontamination methods bring unacceptable changes in the nutritional, sensory, and functional qualities of food and produce adverse toxic residues (Pitt and Hockings, 2009). The physical decontamination methods are most potent due to its spontaneous and strong effects. Particularly, radiation treatment is substantially efficient decontamination technique. The WHO and FDA of the United Nations have recognized that under well-established Good Manufacturing Practices (GMP) the application of γ-radiation in a low dose for disinfestations and enhancement of shelf life of agricultural commodities is nutritionally adequate and acceptable (Calado et al., 2014). Thus, a combination of biological and physical decontamination methods is likely to be a safe and much effective in controlling the fungal growth and mycotoxins content.

To the best of our knowledge, the combinational inhibitory action of essential oil and γ-radiation on the growth rate, production of DON and ZEA by *F. graminearum* has been not reported earlier. Therefore, the present study was aimed to establish the combinational inhibitory effect of *Hedychium spicatum* L. essential oil (HSEO) and radiation for the aforementioned purposes. The *H. spicatum* is a hardy and small perennial plant that belong to family Zingiberaceae and popularly known as “perfume ginger” or “spiked ginger lily” (Joshi et al., 2008). The HSEO is a rich source of diverse medicinal compounds, and it is widely used in herbal medicine to treat a variety of ailments, including nausea, stomachache, dysentery, local inflammations, asthma, bad breath, bronchitis, and rheumatic problems (Koul et al., 2005). The HSEO was obtained from rhizomes by hydrodistillation technique and its chemical profile was revealed by GC-MS analysis. The discrete inhibitory action of HSEO and radiation on the growth rate, production of DON, and ZEA by *F. graminearum* was evaluated in maize grains. Their combinational inhibitory action on aforementioned purposes was evaluated by response surface model adopting central composite design (CCD) and obtained model was validated experimentally.

## Materials and methods

### Chemicals and reagents

Sabouraud dextrose agar (SDA), Sabouraud dextrose broth (SDB), and peptone were obtained from HiMedia (Mumbai, India). Certified standards of DON and ZEA were obtained from Sigma-Aldrich (Bengaluru, India). Immunoaffinity columns specific for DON and ZEA were procured from Vicam (Waters business, USA). The other chemicals used in the study were analytical grade and obtained from Merck Millipore (Bengaluru, India).

### Fungi cultural conditions

*Fusarium graminearum* (MTCC 1893) capable for the synthesis of DON and ZEA was obtained from Microbial Type Culture Collection and Gene Bank (MTCC), India, and grown on SDA for 7 days at 28°C. The fungal spores were collected by soft scrape in sterile peptone solution supplemented with 0.01% Tween 80. The spore number was counted by hemocytometer and their number was set to 1 × 10^6^ spores per mL.

### Collection and characterization of *H. spicatum* L. essential oil

#### Plant material collection and essential oil extraction

The rhizomes of *H. spicatum* were obtained from agricultural field, Ooty, Tamil Nadu, India. Plants were dried under shade for 2 weeks at 27 ± 2°C and ground to a fine powder (voucher no: PEO 37). The essential oil was extracted from 250 g of fine powder by hydrodistillation technique using Clevenger-type apparatus as per the procedure of European Pharmacopoeia (Council of Europe, 1997). The collected essential oil was dried over anhydrous sodium sulfate to get rid of moisture and safeguarded in an amber glass vial at 4°C for further analysis.

#### Chemical characterization of HSEO by GC-MS

The chemical composition of HSEO was revealed by GC–MS analysis using PerkinElmer Clarus 600 C (PerkinElmer, Inc., Waltham, USA) equipped with DB-5MS column (30 m × 0.25 mm; 0.25 μm film thickness) as per our previous reported methodology (Kalagatur et al., 2015). The chemical constituents of HSEO were identified by comparing their mass spectra (MS) with NIST/Wiley library and retention indices (RI) literature of Adams (2007). The quantification (%) of chemical constituents was obtained from the GC peak areas devoid of correction factors.

### Radiation treatment

The radiation treatment was carried out in Gamma radiation chamber-5000 (Freeze Drying and Processing Technology Division, Defense Food Research Laboratory, Mysuru, India) and cobalt 60 was used as a source of γ-radiation with an effective dose frequency of 5.57 kGy per h at 35°C. The absorbed radiation dose was determined by a Ceric-cerous dosimeter that was fixed to the surface of the bottom and top of the test sample. The equivalence of radiation dose was stated as D_max_/D_min_ and it was 1.01 (Reddy et al., 2015).

### Assessment of discrete and combinational inhibitory effects of HSEO and radiation treatments on growth rate, production of DON and ZEA by *F. graminearum* in maize

#### Experimental design

The decisive aim of the present study was to evaluate the discrete and combinational inhibitory effects of HSEO and radiation treatments on the growth rate, production of DON and ZEA by *F. graminearum* in maize grains. The maize grains were obtained from the local agricultural market of Mysuru, Karnataka state, India, and 100 g was packed in plastic bags. The bags were autoclaved at 121°C for 20 min and grains were dried out in a hot air oven at 60°C. The autoclaving process used for removal of background microbial flora could affect the texture of the grains and which could have an impact on the results presented in this study.

The fungal spore suspension of 10 μL (1 × 10^6^ spores per mL) was diluted to 1 mL with sterile peptone water solution and aseptically inoculated to the grain samples and mixed for 15 min at 140–160 rpm in a rotary shaker (Aziz et al., 2007). Following, grain samples were separately exposed to different doses of HSEO and radiation treatments to evaluate their discrete inhibitory effect. The HSEO was directly applied to maize grains and thoroughly vortexed and mixed for 15 min at 140–160 rpm in a rotary shaker as per methodology of Velluti et al. (2003). The combinational exposure of HSEO and radiation treatments with distinctive doses was accomplished by CCD of the response surface methodology (RSM) statistical program adopting State–Ease Design-Expert version 10.0.6.0 software application (Anderson and Whitcomb, 2016). In this study, grain samples initially treated with a distinctive concentration of HSEO were exposed to different doses of radiation treatment as per CCD. The grain samples were incubated for 14 days at water activity of 0.70 and temperature of 28°C. The sample unexposed with HSEO and radiation treatments were referred as a control. Following the incubation period, the growth of fungi, quantification of DON, and ZEA were determined by colony forming units (CFU) and UHPLC, respectively.

The combinational inhibitory effect of HSEO and radiation (independent factors) on the reductions of CFU, DON, and ZEA content (response factors) was assessed by polynomial regression analysis of RSM. The lower and higher range of HSEO (0–3.15 mg/g) and radiation treatments (0–6 kGy) was fixed based on the dose required for complete inhibition of fungal growth and mycotoxins as per the study of their discrete exposure. The coded factors, name, units, range, levels, mean, and standard deviation of independent variables were shown in Supplementary Table 1. The order of experiments within the block was randomized and executed independently for six times. The regression analysis of attained responses i.e. log CFU, DON, and ZEA were assessed by fitting with suitable models represented by the second-order polynomial equation.

$$\begin{array}{l} \text{Y}=\beta_{0}+\overset{n}{\underset{i=1}{\sum }}\beta_{\text{i}}{\text{x}}_{\text{i}}+\overset{\text{n}}{\underset{i=1}{\sum }}\beta_{\text{ii}}{\text{x}}_{\text{i}}^{2}+\overset{n}{\underset{\text{i}\neq \text{j}=1}{\sum }}\beta_{\text{ii}}{\text{x}}_{\text{i}}x_{ij} \end{array}$$

where, “0” was the value of the fitted response at the center point of the design; i, ii, and ij were the linear, quadratic and cross-product (interaction effect) regression terms, respectively and “n” denoted the number of independent variables.

#### Determination of fungal growth by dilution plating technique

The fungal growth in maize grains was determined as per methodology of Aziz et al. (2007) with minor modifications. Following the incubation period, 10 g of maize grains were collected from the plastic bags and suspended in 90 mL of sterile peptone water and mixed for 15 min at 140 – 160 rpm in a rotary shaker. The decimal dilutions were prepared in sterile peptone water and spread plated on SDA plates and incubated at 28°C for 3 days. The fungal growth was determined in colony forming units (CFU) and results were expressed in log CFU/g.

#### Quantification of DON and ZEA by UHPLC

Following the incubation period, 25 g maize grains were collected from the test samples and ground to a fine powder under aseptic conditions and suspended in 250 mL solution of acetonitrile and water (v/v, 6:4). The blend was mixed for 15 min at 140–160 rpm and the supernatant was collected by centrifugation at 6000 rpm for 5 min. The supernatant was subjected to clean-up with immunoaffinity columns of DON and ZEA in according to the instructions of the manufacturer (Vicam, Waters business, USA). The quantification DON and ZEA were done as per our previous reported methodology (Mudili et al., 2014; Kalagatur et al., 2015) using UHPLC system (Nexera, Shimadzu, Japan). The quantification of DON and ZEA was deducted from their respective standard calibration curve and concentration was expressed in μg/g.

### Statistical analysis

The experiments were performed in six independent replicates. The statistical analysis was done according to the one-way ANOVA and significant differences were determined by Tukey's post hoc multiple comparison test and value of *p* < 0.05 was considered significant. The statistical analysis and graphical illustrations were attained adopting the software program GraphPad Prism trial version 7 (GraphPad Software, Inc., USA). The statistical analysis for the optimization of RSM was done by CCD following the State–Ease Design-Expert trial version 10 software program (Stat-Ease, Inc., Minneapolis, USA). The responses of the polynomial regression at a significance level of *p* < 0.05 were considered to design statistical model (Anderson and Whitcomb, 2016). The accuracy of the model was evaluated by measuring the coefficient of determination (*R*^*2*^) and lack of fit.

## Results and discussion

### Chemical composition of HSEO

In the present study, the chemical composition of HSEO was revealed by GC-MS and a total of 48 compounds were identified constituting to 96.84% of total weight (Table 1). The major compounds were 1,8-cineole (23.15%), linalool (12.82%), β-pinene (10.06%), γ-terpinene (8.16%), terpinolene (5.04%), α-terpinene (3.81%), and α-terpineol (3.35%). In our study, β-eudesmol, furanoid, β-himachalene, hedycaryol, eremoligenol, agarospirol, 8-epi-β-bisabolol, and α-cadinol were not detected in accordance with the previous reports of Joshi et al. (2008) and Sabulal et al. (2007). While, diverse compounds, such as terpinolene, α-terpinyl acetate, γ-eudesmol, and γ-muurolene were identified in our study. The chemical compounds and their concentration of essential oil depend on the genetics of plant, part of the plant used, geographical origin, nutrients, harvesting time, and analytical method employed (Gobbo-Neto and Lopes, 2007). Therefore, in our study chemical compounds and their concentration values were varied in comparison to the previous reports.

**Table 1:** Chemical composition of *Hedychium spicatum* L. essential oil determined by GC-MS analysis.

**S. No**	**Compound**	RI^*^	**RI^#^**	**Composition (%)**
1	n-Nonane	903	900	0.18
2	Tricyclene	922	921	0.62
3	α-Thujene	925	924	0.05
4	α-Pinene	935	932	1.31
5	Camphene	948	946	0.19
6	Sabinene	970	969	0.34
7	β-Pinene	976	974	10.06
8	β-Myrcene	989	988	0.89
9	α-Phellandrene	1005	1002	2.27
10	α-Terpinene	1017	1014	3.81
11	p-Cymene	1022	1020	0.22
12	Limonene	1025	1024	2.29
13	1,8-Cineole	1028	1026	23.15
14	(E)-β-Ocimene	1044	1044	1.53
15	γ-Terpinene	1057	1054	8.16
16	Terpinolene	1091	1086	5.04
17	Linalool	1098	1095	12.82
18	n-Nonanal	1104	1100	0.22
19	Camphor	1144	1141	0.73
20	δ-Terpineol	1163	1162	2.89
21	Terpinen-4-ol	1175	1174	2.06
22	α-Terpineol	1188	1186	3.35
23	trans-Carveol	1218	1215	0.02
24	p-Cymen-7-ol	1291	1289	0.85
25	δ-Elemene	1337	1335	0.02
26	α-Terpinyl acetate	1348	1346	2.91
27	β-Elemene	1390	1389	0.87
28	Caryophyllene	1418	1417	0.16
29	Santalene	1450	1447	0.90
30	α-Humulene	1453	1452	0.34
31	γ-Muurolene	1481	1478	0.71
32	Germacrene-D	1486	1484	0.28
33	δ-Selinene	1495	1492	0.73
34	α-Muurolene	1502	1500	0.92
35	Germacrene A	1510	1508	0.06
36	γ-Cadinene	1513	1513	0.14
37	Cubebol	1515	1514	0.19
38	δ-Cadinene	1527	1522	0.07
39	cis-Sesquisabinene hydrate	1543	1542	0.53
40	Elemol	1554	1548	0.04
41	(E)-Nerolidol	1563	1561	0.39
42	Spathulenol	1578	1577	0.81
43	Caryophyllene oxide	1585	1582	0.60
44	10-epi-γ-Eudesmol	1625	1622	0.22
45	1-epi-Cubenol	1629	1627	0.37
46	γ-Eudesmol	1634	1630	0.98
47	α-Muurolol	1649	1644	0.70
48	α-Eudesmol	1655	1652	0.85
Total				96.84%

* *Actual retention indices of compounds on DB-5 column*.

# *Retention indices of compounds on DB-5 column in accordance to literature of Adams (2007)*.

### Inhibitory effect of HSEO and radiation treatments on growth rate, production of DON and ZEA by *F. graminearum* in maize

#### Discrete treatment of HSEO and radiation

**Figure 1:**
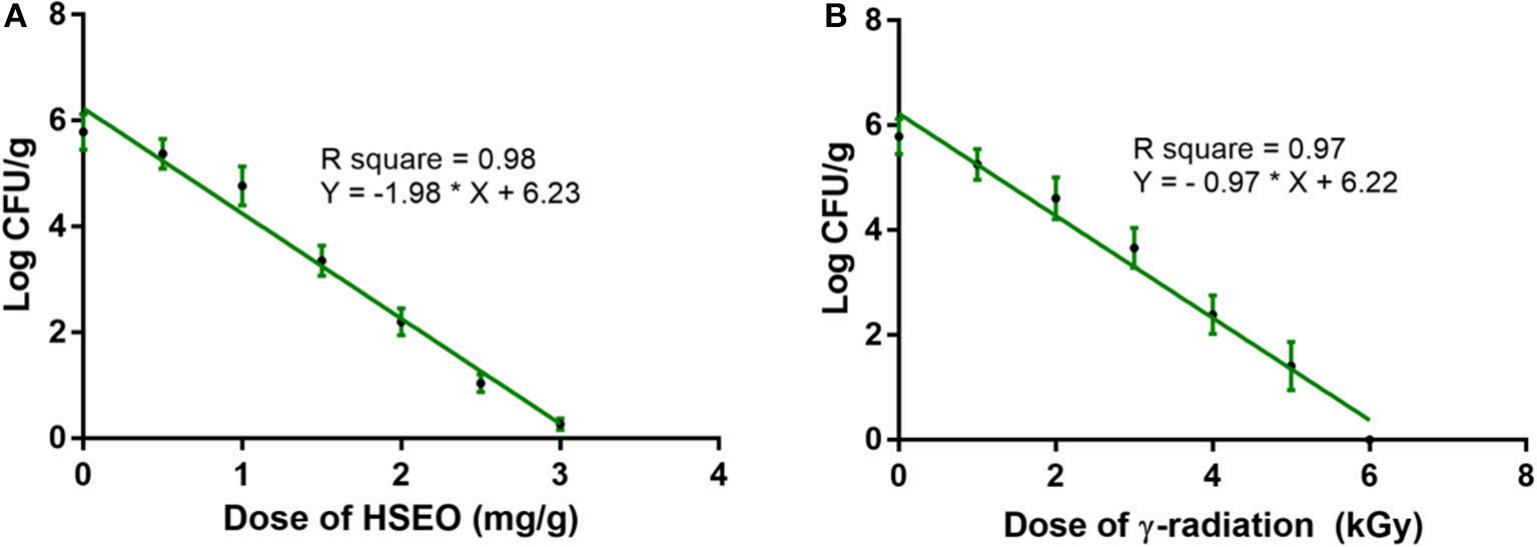
Linear regression curve for growth inhibitory activity of different doses of **(A)** *H. spicatum* L. essential oil (HSEO) and **(B)** radiation treatments on *F. graminearum* in maize. The log CFU/g was declined with the dose of HSEO and radiation. The data was processed by one-way ANOVA following Tukey's test and value of *p* < 0.05 was considered significant.

**Figure 2:**
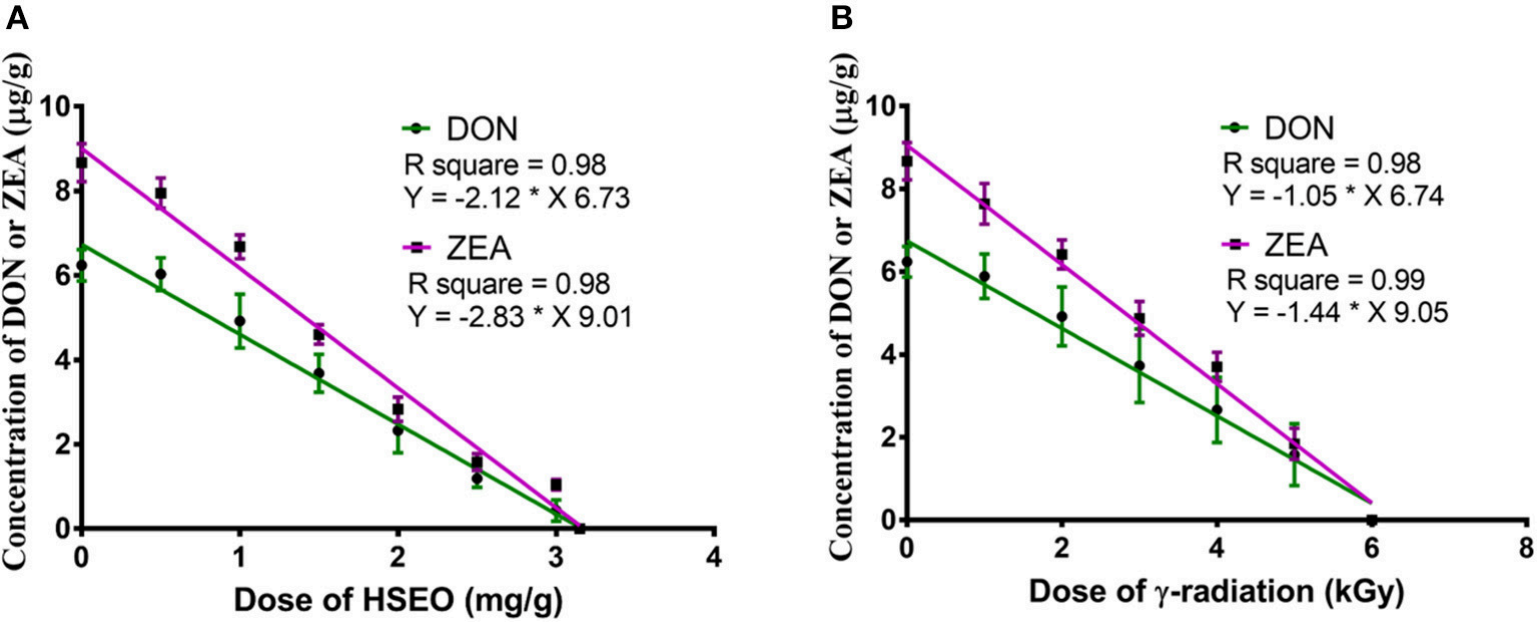
Linear regression curve for dose-dependent effect of **(A)** *H. spicatum* L. essential oil (HSEO) and **(B)** radiation treatments on the reductions of deoxynivalenol (DON) and zearalenone (ZEA) content by *F. graminearum* in maize. The data was analyzed by one-way ANOVA following Tukey's test and value of *p* < 0.05 was considered significant.

In the present study, discrete treatment of HSEO and radiation were effective in reducing the fungal growth (log CFU), production of DON and ZEA by *F. graminearum* in maize grains. A quantity of 5.79 ± 0.33 of log CFU/g, 6.24 ± 0.37 of DON (μg/g), and 8.67 ± 0.45 of ZEA (μg/g) were determined in the control sample. While, log CFU, DON, and ZEA content were reduced in test samples in a dose-dependent way with treatment of HSEO and radiation (Tables 2, 3). The fungal growth, DON, and ZEA content were not detected at 3.15 mg/g of HSEO and 6 kGy of radiation. The linear regression curves for reductions of log CFU, DON, and ZEA content were constructed against different doses of HSEO and radiation (Figures 1, 2). The obtained regression models exhibited the goodness of fit (*R*^*2*^) close to 1 and found statistically significant (*p* < 0.05; Tables 4, 5). The regression models confirmed that reductions of log CFU, DON, and ZEA content by HSEO and radiation were dose-dependent.

**Table 2:** Discrete inhibitory effect of *H. spicatum* essential oil (HSEO) on growth rate (log CFU), production of deoxynivalenol (DON) and zearalenone (ZEA) by *F. graminearum* in maize grains.

**S. No**	**Dose of HSEO (mg/g)**	**Log CFU/g**	**DON (μg/g)**	**ZEA (μg/g)**
1	0 (control)	5.79 ± 0.33^a^	6.24 ± 0.37^a^	8.67 ± 0.45^a^
2	0.5	5.38 ± 0.28^ab^	6.03 ± 0.39^ab^	7.95 ± 0.36^b^
3	1	4.77 ± 0.37^c^	4.92 ± 0.64^c^	6.68 ± 0.28^c^
4	1.5	3.36 ± 0.29^d^	3.68 ± 0.45^d^	4.60 ± 0.23^d^
5	2	2.20 ± 0.26^e^	2.33 ± 0.53^e^	2.83 ± 0.29^e^
6	2.5	1.04 ± 0.17^f^	1.19 ± 0.21^f^	1.58 ± 0.20^f^
7	3	0.27 ± 0.11^g^	0.43 ± 0.25^g^	1.04 ± 0.13^g^
8	3.15	0^gh^	0^gh^	0^h^

*The data was processed by one-way ANOVA following Tukey's test and the columns with same alphabetic letters were not significant (p < 0.05)*.

**Table 3:** Discrete inhibitory effect of radiation treatment on growth rate (log CFU), production of deoxynivalenol (DON) and zearalenone (ZEA) by *F. graminearum* in maize grains.

**S. No**	**Dose of radiation (kGy)**	**Log CFU/g**	**DON (μg/g)**	**ZEA (μg/g)**
1	0 (control)	5.79 ± 0.33^a^	6.24 ± 0.37^a^	8.67 ± 0.45^a^
2	1	5.26 ± 0.30^ab^	5.89 ± 0.54^ab^	7.64 ± 0.49^b^
3	2	4.61 ± 0.40^c^	4.92 ± 0.71^bc^	6.42 ± 0.35^c^
4	3	3.66 ± 0.38^d^	3.73 ± 0.89^d^	4.87 ± 0.41^d^
5	4	2.39 ± 0.37^e^	2.66 ± 0.79^de^	3.70 ± 0.35^e^
6	5	1.41 ± 0.46^f^	1.58 ± 0.75^ef^	1.84 ± 0.38^f^
7	6	0^g^	0^g^	0^g^

*The data was processed by one-way ANOVA following Tukey's test and the columns with same alphabetic letters were not significant (p < 0.05)*.

**Table 4:** Linear regression curve for discrete inhibitory effect of *H. spicatum* essential oil (HSEO) on growth rate (log CFU), production of deoxynivalenol (DON) and zearalenone (ZEA) by *F. graminearum* in maize grains.

	**Log CFU/g**	**DON (μg/g)**	**ZEA (μg/g)**
**BEST-FIT VALUES** ± **SE**			
Slope	−1.98 ± 0.12	−2.12 ± 0.10	−2.83 ± 0.14
Y-intercept	6.23 ± 0.22	6.73 ± 0.20	9.01 ± 0.29
X-intercept	3.14	3.16	3.17
1/slope	−0.50	−0.46	−0.35
**95% CONFIDENCE INTERVALS**			
Slope	−2.31 to −1.66	−2.37 to −1.88	−3.19 to −2.48
Y-intercept	5.65 to 6.82	6.23 to 7.23	8.29 to 9.72
X-intercept	2.86 to 3.50	2.97 to 3.39	2.96 to 3.43
**GOODNESS OF FIT**			
R square	0.98	0.98	0.98
Sy.x	0.33	0.30	0.44
**IS SLOPE SIGNIFICANTLY NON-ZERO?**			
*F*	248.3	446.8	379.8
DFn, DFd	1, 6	1, 6	1, 6
*P*-value	<0.005	<0.005	<0.005
Deviation from zero?	Significant	Significant	Significant
Equation	Y = −1.98^*^X + 6.23	Y = −2.12^*^X + 6.73	Y = −2.83^*^X + 9.01

**Table 5:** Linear regression curve for discrete inhibitory effect of radiation treatment on growth rate (log CFU), production of deoxynivalenol (DON) and zearalenone (ZEA) by *F. graminearum* in maize grains.

	**Log CFU/g**	**DON (μg/g)**	**ZEA (μg/g)**
**BEST-FIT VALUES** ± **SE**			
Slope	−0.97 ± 0.06	−1.05 ± 0.06	−1.44 ± 0.06
Y-intercept	6.22 ± 0.23	6.74 ± 0.23	9.05 ± 0.2
X-intercept	6.38	6.38	6.28
1/slope	−1.02	−0.94	−0.69
**95% CONFIDENCE INTERVALS**			
Slope	−1.14 to −0.80	−1.22 to −0.88	−1.60 to −1.27
Y-intercept	5.63 to 6.82	6.13 to 7.35	8.46 to 9.64
X-intercept	5.80 to 7.17	5.82 to 7.10	5.88 to 6.77
**GOODNESS OF FIT**			
R square	0.97	0.98	0.99
Sy.x	0.34	0.34	0.33
**IS SLOPE SIGNIFICANTLY NON-ZERO?**			
*F*	229.5	260.4	515.4
DFn, DFd	1, 5	1, 5	1, 5
*P*-value	<0.005	<0.005	<0.005
Deviation from zero?	Significant	Significant	Significant
Equation	Y = −0.97^*^X + 6.22	Y = −1.05^*^X + 6.74	Y = −1.44^*^X + 9.05

Best of our knowledge, till the date, the fungicidal activity of HSEO on *F. graminearum* was not reported, and this is the first report. On the other hand, Pawar and Thaker (2006) has determined the antifungal activity of HSEO on *Aspergillus niger* by a zone of inhibition assay as 8 mm. The growth inhibitory activity of radiation treatment on *F. graminearum* was less investigated. In support of our study, very few reports are available on the application of radiation treatment in the management of *F. graminearum* in food and feed matrices. Aziz et al. (2007) have reported the complete inhibition of *Fusarium* spp. growth at 4 kGy in barley and 6 kGy in wheat and maize grains. On the other hand, Ferreira-Castro et al. (2007) and Lima et al. (2011) have documented that high dose of 10 kGy radiation treatment was required for complete reduction of *Fusarium* spp. in maize and cowpea bean grains, respectively. The radiation dose required for reduction of fungal growth depends on certain factors, such as the type of species, population number, intensity of pigmentation, water content, genetics, and induction of protective enzymes, which play a major role in the regaining of the damage induced by radiation (Jeong et al., 2015). In the present study, the background microbial flora of maize grains was removed by autoclave sterilization and which could affect the texture of the grains and have an impact on the results. Therefore, the differences in the radiation dose required for complete reduction of fungal growth were noticed in present and previous studies.

#### Combinational treatment of HSEO and radiation

Radiation process is measured as an ideal and beneficial decontamination technique at a low dose. High radiation dose produces intolerable features and toxic substances in food, such as structural and chemical changes, nutritional and sensory loss, undesirable odor and flavor, and rancidity (Calado et al., 2014). Therefore, minimally radiation processed foods are highly preferred and acceptable by consumer and regulatory bodies. In this context, food industry and food technologists have made many efforts through last decade to reduce the efficient radiation dosage rate and augment the decontamination efficiency of radiation. Currently, combining radiation with other decontamination agents, such as essential oils, chemical preservatives, and modified atmospheres is an effective and innovative way to improve the decontamination efficiency of radiation (Ghosh et al., 2017; Sirocchi et al., 2017; Wilson et al., 2017). The combinational approach reduces the possible means radiation dose required for decontamination of microbial flora and could promote food safety at low radiation dosage.

The combinational inhibitory effect of HSEO and radiation treatments on the fungal growth (log CFU), production of DON and ZEA by *F. graminearum* in maize grains were executed following the CCD of RSM statistical program (Wu et al., 2017). The RSM is a collection of statistical and mathematical techniques, and one of the widely used applications in the design, development, and invention of new process or products, and improvement of an existing process or product designs in food science and technology. The RSM requires only a minimal number of experiments between input variables that potentially influence performance measures or quality characteristics of the product or process and allow to identify breakthrough productive information by means of reducing cost, errors, and disturbance. The relations between the input factors and responses were hypothesized or assessed by choosing a model (Whitcomb and Anderson, 2004).

The actual or obtained response of 13 CCD experiments of the present study was provided in Table 6. The data was analyzed by second-order polynomial regression analysis employing the software Design Expert (version 10.0.6) to optimize the response surface models.

**Table 6:** Central composite design (CCD) for combinational inhibitory action of *H. spicatum* essential oil (HSEO) and radiation treatments (independent factors) on the reductions of log CFU, DON, and ZEA (responses).

**Run order**	**Independent factors**		**Actual responses**		
	**A: HSEO (mg/g)**	**B: radiation (kGy)**	**Log CFU/g**	**DON (μg/g)**	**ZEA (μg/g)**
1	0.25 (−1)	5.52 (1)	1.02 ± 0.33	1.19 ± 0.23	1.56 ± 0.41
2	1.57 (0)	3(0)	1.07 ± 0.18	1.26 ± 0.47	1.71 ± 0.67
3	0.25 (−1)	0.47 (−1)	5.42 ± 0.37	5.97 ± 0.54	7.68 ± 0.84
4	1.57 (0)	3 (0)	0.84 ± 0.35	1.14 ± 0.34	1.32 ± 0.28
5	1.57 (0)	3 (0)	0.92 ± 0.49	1.31 ± 0.18	1.58 ± 0.55
6	1.57 (0)	3 (0)	0.94 ± 0.32	1.27 ± 0.48	1.83 ± 0.28
7	1.57 (0)	3 (0)	0.81 ± 0.20	1.02 ± 0.29	1.64 ± 0.40
8	1.57 (0)	6 (2)	0.00	0.00	0.00
9	2.89 (1)	0.47 (−1)	0.84 ± 0.31	1.12 ± 0.20	1.47 ± 0.25
10	0 (−2)	3 (0)	3.79 ± 0.58	4.12 ± 0.42	5.38 ± 0.45
11	2.89 (1)	5.52 (1)	0.00	0.00	0.00
12	3.15 (2)	3 (0)	0.00	0.00	0.00
13	1.57 (0)	0 (−2)	3.12 ± 0.66	3.48 ± 0.89	4.71 ± 0.38

**Figure 3:**
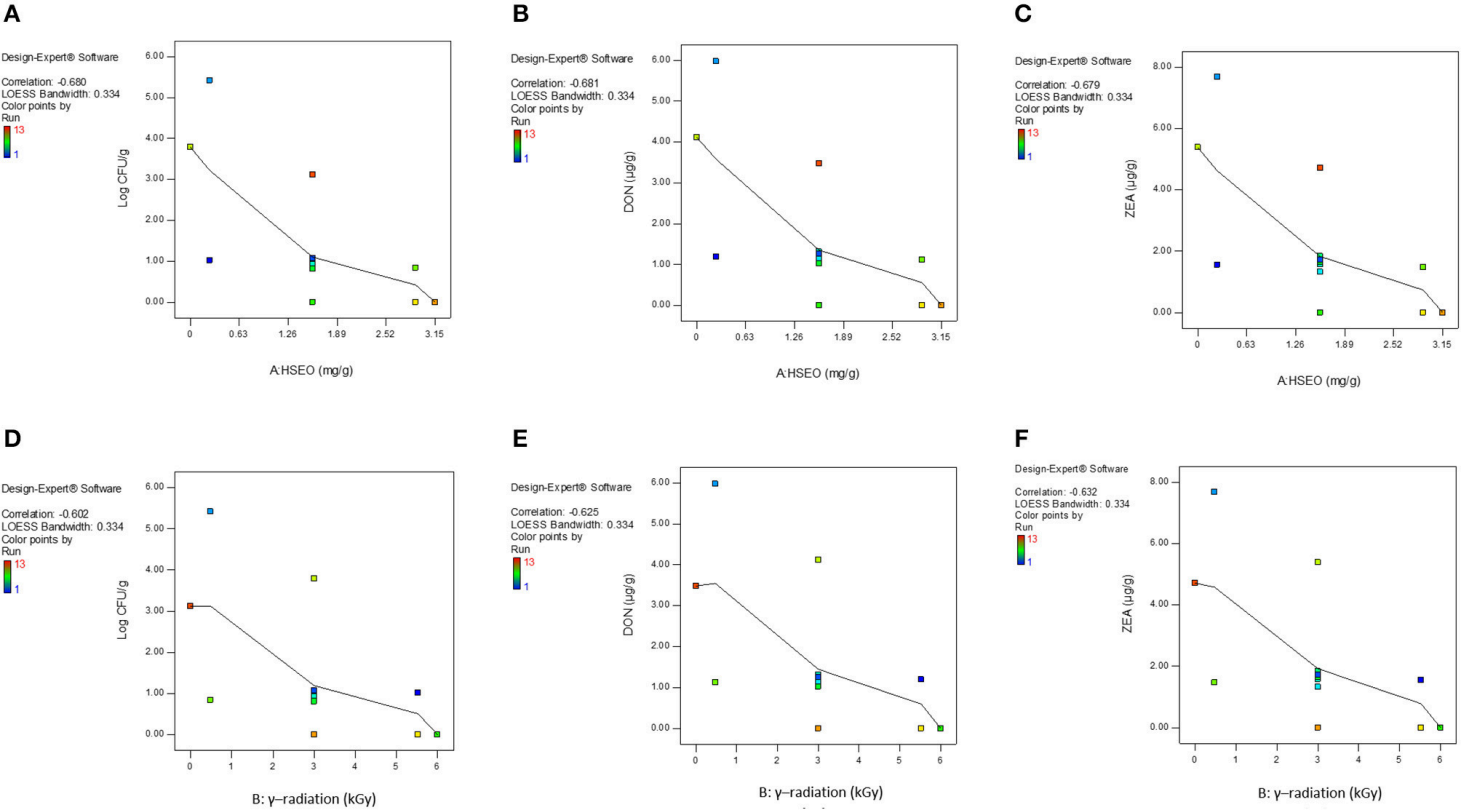
Correlation matrix plots of HSEO **(A–C)** and radiation treatments **(D–F)** to accomplish their suitability for responses: log CFU, DON, and ZEA.

The correlation matrix of regression coefficients of independent factors was correlated with one another on a scale of perfect negative correlation (−1) to perfect positive correlation (+1) as per Pearson's correlation coefficients to conclude their appropriateness. The perfect negative correlated matrix was appropriate and agrees to the individual effect of independent factors on responses (Anderson and Whitcomb, 2016). In the present study, a perfect negative correlation matrix was observed for both independent factors (Figure 3). A perfect negative correlation matrix of − 0.680 and − 0.602 (log CFU), − 0.681 and − 0.625 (DON), and − 0.679 and − 0.632 (ZEA) were observed for HSEO and radiation treatments, respectively. It indicated that independent factors were independently effective in reducing the log CFU, DON, and ZEA. These results were in accordance with the assessment of the discrete inhibitory effect of HSEO and radiation treatments on the reductions of log CFU, DON, and ZEA content (Figures 1, 2). Therefore, the combinational assessment of HSEO and radiation treatments for the aforementioned purposes were well appropriate and could reveal newer insights. Consequently, combinational treatments of HSEO and radiation were highly effective in reducing the log CFU, DON, and ZEA content in maize grains. The complete reductions of fungal growth, DON, and ZEA content were noticed at combination of 1.89 mg/g of HSEO and 4.12 kGy of radiation treatments (**Table 11**). The dose of HSEO (1.89 mg/g) and radiation (4.12 kGy) required for complete reductions of fungal growth, DON, and ZEA content were much less in combinational treatment compared to discrete treatments of HSEO (3.15 mg/g) and radiation (6 kGy).

The obtained CCD results of the RSM study concluded that quadratic model was well appropriate for all the responses (log CFU, DON, and ZEA). The coefficient of independent variables in terms actual factors for second-order polynomial equation designed for the responses attained as below:

$$\begin{array}{l} \text{Log CFU}/\text{g}=+\;6.87\;-\;2.98*\text{HSEO }-\;1.27*\text{radiation }\\ +\;0.26*\text{HSEO}*\text{radiation}+\;0.34*{\text{HSEO}}^{2}\;\\ +\;0.05*{\text{radiation}}^{2} \end{array}$$

$$\begin{array}{l} \text{DON }\left(\mu \text{g/g}\right)=+\;7.42\;-\;3.03*\text{HSEO }-\;1.32*\text{radiation }\\ +\;0.27*\text{HSEO}*\text{radiation }+\;0.31*{\text{HSEO}}^{2}\;\\ +\;0.05*{\text{radiation}}^{2} \end{array}$$

$$\begin{array}{l} \text{ZEA }\left(\mu \text{g/g}\right)=+\;9.61\;-\;3.81*\text{HSEO }-1.72*\text{radiation }\\ +\;0.34*\text{HSEO}*\text{radiation }+\;0.38*{\text{HSEO}}^{2}\;\\ +\;0.06*{\text{radiation}}^{2} \end{array}$$

The significance of the second-order polynomial model was evaluated by analysis of variance (ANOVA) and coefficient of determination (*R*^*2*^). The ANOVA results for the fitted quadratic polynomial models of log CFU, DON, and ZEA were presented in Tables 7, 8, 9, respectively. For any of the terms in the model, a greater *F*-value and smaller *p*-value would indicate a more significant effect on the respective response variables (Atkinson and Donev, 1992). The present model has larger *F*-value of 251.61 (log CFU), 335.88 (DON), and 229.45 (ZEA) and the associated *p*-value < 0.0001 and implied that the optimized models were very significant. Furthermore, *F*-value and *p*-value of the lack of fit were 4.44 and 0.0918 for log CFU, 2.37 and 0.2120 for DON, 2.24 and 0.2255 for ZEA, respectively (Table 10). Which indicated that lack of fit was not significant and 9.18%, 21.20%, and 22.55% chance could occur due to noise in log CFU, DON, and ZEA, respectively. The goodness of fit of the models was judged by estimating the coefficient of determination (*R*^*2*^). The value of *R*^*2*^ for log CFU, DON, and ZEA were observed as 0.9945, 0.9958, and 0.9939, respectively. Which implies that 99.45%, 99.58%, and 99.39% of the variations could be explained by the fitted models of log CFU, DON, and ZEA, respectively. For a good statistical model, the predicted R-squared value should be close to adjusted R-squared value and the obtained differences in all the responses of the present study were appropriate (Table 10). Also, adequate precision should be >4.0 is desirable for the significant model and adequate precision of 52.343 (log CFU), 60.519 (DON), and 50.190 (ZEA) in the present study indicates an adequate signal and the obtained models were well appropriate to navigate the design space (Table 10).

**Table 7:** ANOVA for log CFU/g response surface quadratic model.

**Source**	**Sum of squares**	**Degree of freedom (df)**	**Mean square**	***F-*value**	***p*-value Prob > *F***
Model	32.18	5	6.44	251.61	0.0918 significant
A-HSEO	14.96	1	14.96	584.89	<0.0001
B-Radiation	11.73	1	11.73	458.67	<0.0001
AB	3.17	1	3.17	123.87	<0.0001
A^2^	1.56	1	1.56	60.84	0.0001
B^2^	0.57	1	0.57	22.11	0.0022
Residual	0.18	7	0.026		
Lack of fit	0.14	3	0.046	4.44	0.0918 not significant
Pure error	0.041	4	0.010		
Cor total	32.36	12			

**Table 8:** ANOVA for DON (μg/g) response surface quadratic model.

**Source**	**Sum of squares**	**Degree of freedom (df)**	**Mean square**	***F-*value**	***p*-value Prob > *F***
Model	37.67	5	7.53	335.88	<0.0001 significant
A-HSEO	17.53	1	17.53	781.33	<0.0001
B-Radiation	14.76	1	14.76	657.91	<0.0001
AB	3.35	1	3.35	149.30	<0.0001
A^2^	1.38	1	1.38	61.36	0.0001
B^2^	0.49	1	0.49	21.84	0.0023
Residual	0.16	7	0.022		
Lack of fit	0.10	3	0.033	2.37	0.2120 not significant
Pure error	0.057	4	0.014		
Cor total	37.83	12			

**Table 9:** ANOVA for ZEA (μg/g) response surface quadratic model.

**Source**	**Sum of squares**	**Degree of freedom (df)**	**Mean square**	***F-*value**	***p*-value Prob > *F***
Model	63.36	5	12.67	229.45	<0.0001 significant
A-HSEO	29.40	1	29.40	532.25	<0.0001
B-Radiation	25.48	1	25.48	461.39	<0.0001
AB	5.41	1	5.41	97.88	<0.0001
A^2^	1.98	1	1.98	35.84	0.0005
B^2^	0.83	1	0.83	15.04	0.0061
Residual	0.39	7	0.055		
Lack of fit	0.24	3	0.081	2.24	0.2255 not significant
Pure error	0.14	4	0.036		
Cor total	63.75	12			

**Table 10:** Sequential model and regression coefficients of optimized designs.

**Term model**	**Responses**		
	**Log CFU/g**	**DON (μg/g)**	**ZEA (μg/g)**
*F*-Value	251.61	335.88	229.45
*P* > F	<0.0001	<0.0001	<0.0001
Mean	1.44	1.68	2.22
Standard deviation	0.16	0.15	0.24
C V %	11.08	8.90	10.58
R squared	0.9945	0.9958	0.9939
Adjusted R squared	0.9905	0.9929	0.9896
Predicted R squared	0.9664	0.9771	0.9673
Adequate precision	52.343	60.519	50.190
Model	Quadratic	Quadratic	Quadratic

**Figure 4:**
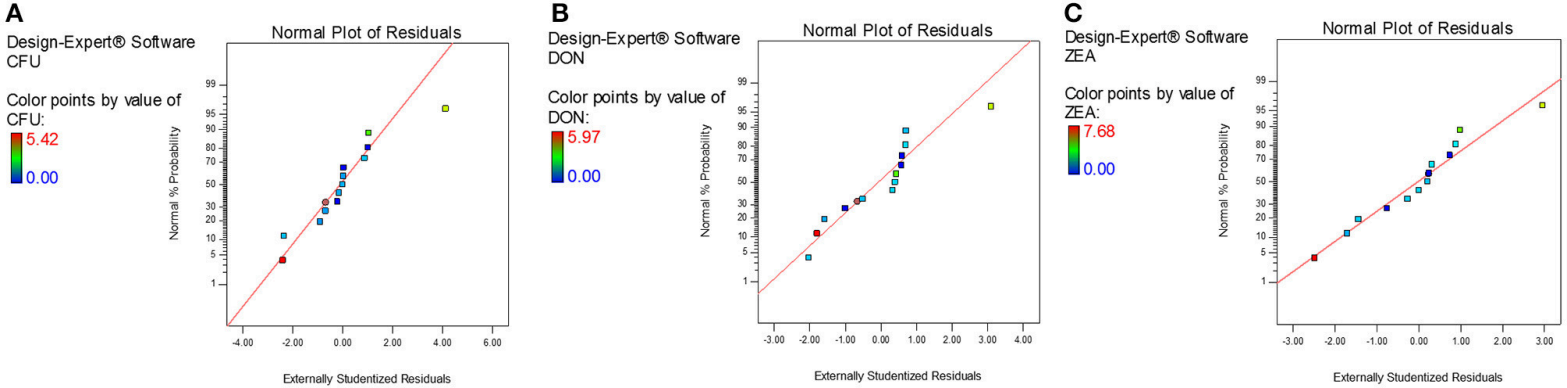
Normal plot of residuals for combinational inhibitory effect of *H. spicatum* L. essential oil (HSEO) and radiation treatments on **(A)** log CFU/g (fungal growth), production of **(B)** DON (μg/g) and **(C)** ZEA (μg/g) by *F. graminearum* in maize grains.

**Figure 5:**
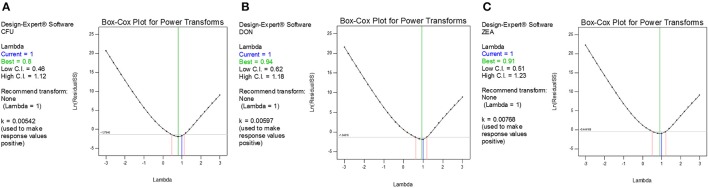
Box-Cox plot for determination of the best power-transformed response surface model: **(A)** log CFU, **(B)** DON, and **(C)** ZEA.

**Figure 6:**
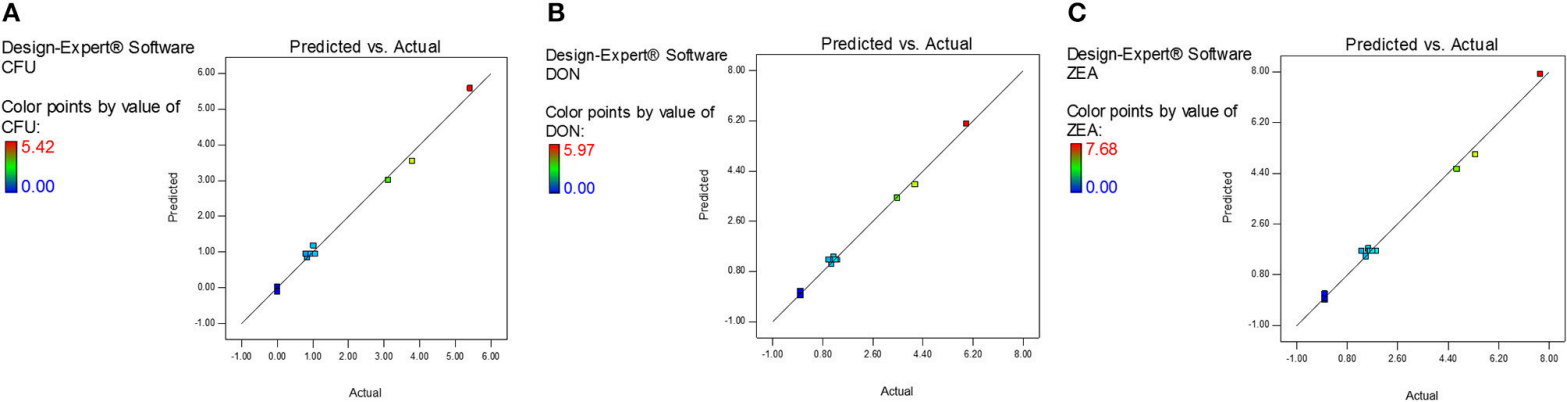
Predicted vs. actual plots for combinational inhibitory effect of *H. spicatum* L. essential oil (HSEO) and radiation treatments on responses: **(A)** log CFU, **(B)** DON, and **(C)** ZEA.

**Figure 7:**
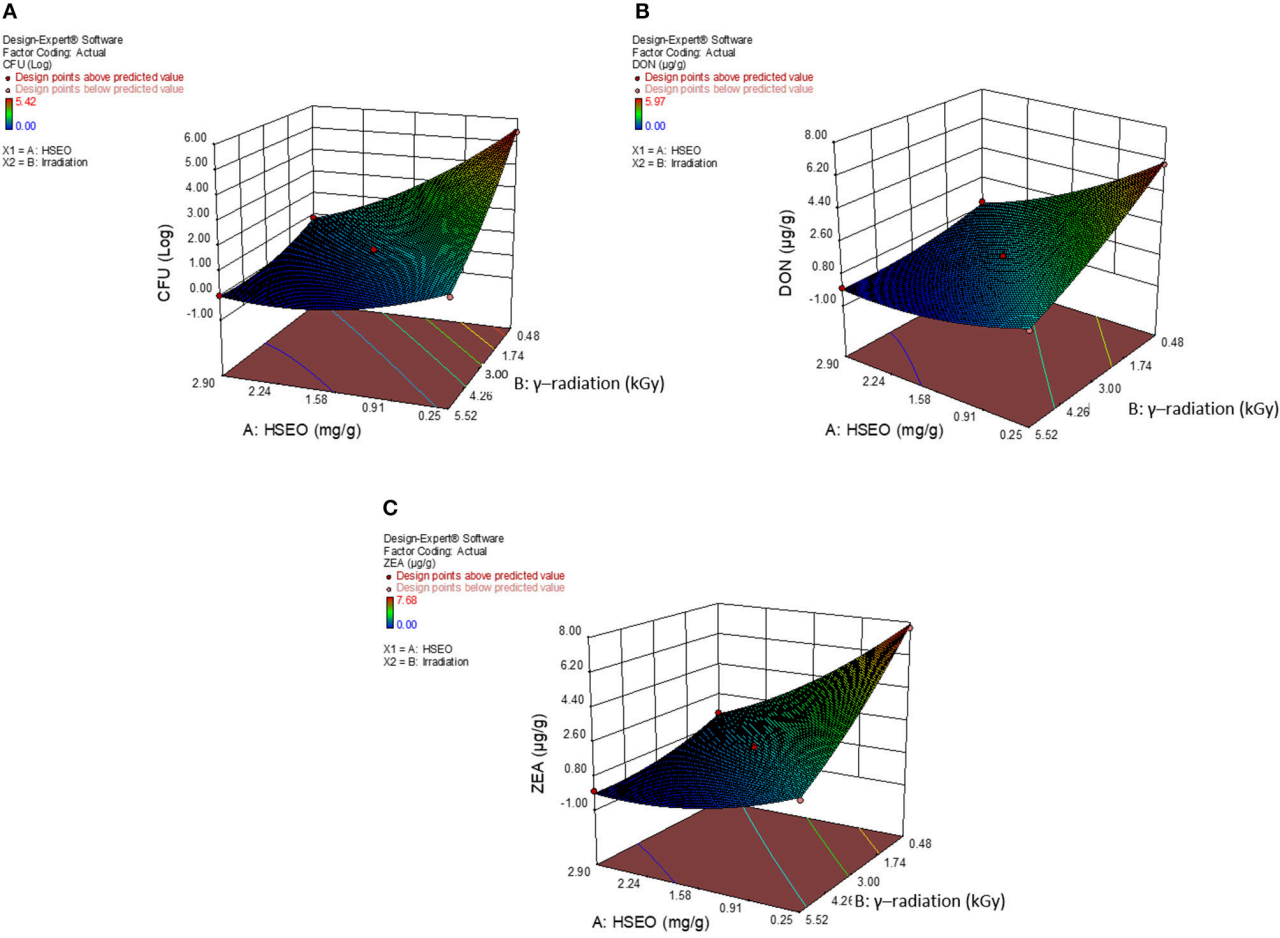
3D-response surface plot for combinational inhibitory effect of *H. spicatum* L. essential oil (HSEO) and radiation treatments on **(A)** log CFU/g (fungal growth), production of **(B)** DON (μg/g) and **(C)** ZEA (μg/g) by *F. graminearum* in maize grains.

The diagnostic and correlation plots were judged to endorse the obtained significance of ANOVA. The normal plot residuals of normal % probability vs. externally studentized residuals were constructed to illustrate the accuracy of optimized design (Figure 4). The distribution of externally studentized residuals was satisfactorily followed the normal distribution. Which indicated that residuals plots follow linear behavior and the predicted model is accurate (Myers et al., 2016). A Box-Cox plot for power transforms of responses were considered to determine the most appropriate power law transformation to fit the responses. The best recommended transform (λ) of 0.80, 0.94, and 0.91 were observed for log CFU, DON, and ZEA, respectively (Figure 5). The obtained λ values were close to the current value of 1 for none and which indicates that responses were well fitted by the optimized design (Box and Cox, 1964). The optimized designs were assessed to examine the correlation between the actual and predicted responses (Figure 6). The obtained data points were close to the straight line and presented a good correlation coefficient (*R*^*2*^). The correlation curves suggested a high degree correlation of 0.9945, 0.9958, and 0.9939 between the actual and predicted values of the log CFU, DON, and ZEA, respectively and concluded that the fundamental assumptions of the analysis were well appropriate. The fitted polynomial equation was expressed as 3D surface plots to illustrate the interactive effects of the independent variables on the responses and to deduce optimum conditions (Figure 7). In conclusion, appropriateness of the optimized model for predicting the optimum responses was verified with suggested optimum conditions. The obtained actual values of suggested experiments were found in agreement with the predicted values (Table 11). The complete reductions of fungal growth (log CFU), DON, and ZEA content were noticed in maize grains at combinational treatment of 1.89 mg/g of HSEO and 4.12 kGy of radiation. These results suggested that the designed quadratic models for reductions of log CFU, DON, and ZEA content in maize grains were statistically significant and could adequately represent the real interdependence of factors chosen (HSEO and radiation).

**Table 11:** Assessment of proposed predicted values of design with actual values of experiment to maximize the inhibition of growth rate, DON, and ZEA by *F. graminearum* in maize grains.

**S. No**	**Independent factors**		**Responses**					
			**Predicted**			**Actual**		
	**A: HSEO (mg/g)**	**B: Radiation (kGy)**	**Log CFU/g**	**DON (μg/g)**	**ZEA (μg/g)**	**Log CFU/g**	**DON (μg/g)**	**ZEA (μg/g)**
1	1.50	3.45	0.77 ± 0.26	1.01± 0.06	1.38 ± 0.10	0.84 ± 0.14	1.09 ± 0.22	1.41 ± 0.28
2	0.25	0.47	5.58 ± 0.50	6.10 ± 0.12	7.92 ± 0.19	5.71 ± 0.39	6.17 ± 0.53	8.08 ± 0.71
3	0.84	1.63	3.03 ± 0.27	3.44 ± 0.06	4.52 ± 0.10	2.92 ± 0.28	3.61 ± 0.41	4.29 ± 0.47
4	1.12	3.95	0.95 ± 0.25	1.20 ± 0.06	1.60 ± 0.10	0.81 ± 0.21	1.05 ± 0.34	1.22 ± 0.15
5	1.57	2.68	1.11 ± 0.26	1.40 ± 0.06	1.88 ± 0.10	1.19 ± 0.17	1.64 ± 0.22	1.79 ± 0.12
6	0.79	2.97	2.04 ± 0.26	2.37 ± 0.06	3.12 ± 0.10	1.87 ± 0.33	2.44 ± 0.39	3.51 ± 0.44
7	1.89	1.03	1.69 ± 0.29	2.03 ± 0.07	2.73 ± 0.11	1.80 ± 0.26	2.16 ± 0.41	2.98 ± 0.34
8	0.79	3.87	1.18 ± 0.25	1.44 ± 0.06	1.91 ± 0.10	1.24 ± 0.22	1.52 ± 0.24	1.87 ± 0.21
9	0.43	1.70	3.80 ± 0.32	4.23 ± 0.08	5.51 ± 0.12	3.92 ± 0.47	4.16 ± 0.32	5.67 ± 0.64
10	1.82	3.63	0.41 ± 0.25	0.62 ± 0.06	0.86 ± 0.10	0.58 ± 0.11	0.74 ± 0.12	1.03 ± 0.19
11	1.89	4.12	0	0	0	0	0	0

The present study concluded that combinational exposure of HSEO and radiation on the reductions of fungal growth, DON, and ZEA content by *F. graminearum* in maize grains was highly efficient compared to their discrete exposure. In combinational approach, radiation dose required for complete reductions of fungi and mycotoxins were reduced from 6 kGy (discrete treatment) to 4.12 kGy. Which indicated that radiation treatment with the combination of HSEO was highly efficient and minimally radiation processed compared to unaccompanied treatment of radiation. However, structural, chemical and nutritional changes of maize grains were not determined in the present study. In support of our study, earlier reports demonstrated that essential oils with a combination of modified atmosphere packaging, mild heat treatment, and radiation were highly effective and safe in maintaining the microbiological safety of the food (Hossain et al., 2014). Several studies noticed that antifungal activity of essential oils is reliant on chemical constituents and their synergistic action (Hyldgaard et al., 2012; da Cruz Cabral et al., 2013). In our study, varieties of chemical compounds were noticed in HSEO (Table 1) and the attributed antifungal activity of HSEO against *F. graminearum* could be due to individual and combination of action of 1,8-cineole, linalool, β-pinene, limonene, germacrene-D, γ-terpinene, terpinolene, α-terpinene, and α-terpineol (Velluti et al., 2003; Van Vuuren and Viljoen, 2007; Deba et al., 2008; Chee et al., 2009; Vilela et al., 2009; Gao et al., 2016). These compounds could alter the integrity of cell membrane and disturb the membrane fluidity, osmotic balance, enzymatic functions, and leak cytoplasmic contents of the cell. These cascade of events stops the ATP synthesis, growth and proliferation of fungi, and activates the caspase-mediated apoptotic death by releasing the mitochondrial cytochrome c and death receptors (Ramsdale, 2008; Usta et al., 2009; Tian et al., 2012; Chen et al., 2014). In case of radiation treatment, radiolysis of cellular water of organisms produces positive-charged water radicals (H_2_O^+^) and negative-charged free electrons (e^−^), and a series of cross-combination and recombination reactions generate highly reactive molecules (de Campos et al., 2004; Le Caër, 2011). These molecules attack and cleave hydrogen from sugar and bases of nuclear material, and destruct the other cellular components, i.e., protein and lipids and even damage the integrity of cellular membranes. This process inhibits spore germination and biomass production and promotes the death of fungi (Calado et al., 2014).

Henceforth, combined treatments of HSEO and radiation presented a superior antifungal efficiency on *F. graminearum* than the discrete treatment of HSEO or radiation alone. Moreover, many reports showed that the combinational treatment of essential oils with radiation up to a dose of 10 kGy do not produce any significant changes in the quality and quantity of essential oils and not produce any toxic residues (Chatterjee et al., 2000; Haddad et al., 2007). Consequently, functional features of essential oils do not effect on exposure to radiation and acceptable by Institute of Food Science & Technology (IFST), WHO, FAO, International Atomic Energy Agency (IAEA), and consumer (Lacroix and Ouattara, 2000; IAEA, 2001; Maherani et al., 2016). Henceforth, the proposed novel combinational exposure of HSEO and radiation could be highly acceptable and efficient for reductions of fungal growth and mycotoxins in food and feed matrices and thereby support in obtaining the highly microbiological safe food products. Though, a greater understanding of the combinational antifungal activity of essential oils and radiation is required at the cellular and molecular level to exploit underlying the molecular death machinery.

## Conclusion

In the present study, the discrete and combinational inhibitory effects of HSEO and radiation treatments on growth, production of DON and ZEA by *F. graminearum* in maize grains were assessed under laboratory set-ups. The GC-MS analysis of HSEO revealed the presence of 48 compounds constituting to 96.84% of total weight and major compounds were 1,8-cineole, linalool, and β-pinene. The simple linear regression analysis showed that HSEO and radiation treatments were discretely inhibited the fungal growth, DON, and ZEA content in maize grains by the dose-dependent way, and complete inhibition were noticed at 3.15 mg/g of HSEO and 6 kGy of radiation. The combinational inhibitory action of HSEO and radiation treatments on growth, production of DON and ZEA by *F. graminearum* in maize grains was studied by CCD of RSM statistical program. The fungal growth, DON, and ZEA content were not detected at combinational treatment of 1.89 mg/g of HSEO and 4.12 kGy of radiation. The results revealed that combination of HSEO and radiation treatments could reduce the fungal growth, DON, and ZEA content at much lower concentration than their discrete inhibitory dose. The quadratic model was found well appropriate for reductions of fungal growth (log CFU), DON, and ZEA content in maize grains. The optimized design was found statistically significant (*p* < 0.05) with larger *F*-value and adequate precision, and a smaller *p*-value. The actual data points were close to the straight line and presented good correlation regression coefficients (*R*^*2*^) for the responses. In addition, diagnostic and correlation plots, i.e., correlation matrix, normal plot residuals, Box-Cox, and actual vs. predicted plots were confirmed that optimized design was accurate and appropriate. The study concluded that optimized design could adequately represent the real interdependence of factors chosen (HSEO and radiation) for reductions of fungal growth, DON, and ZEA content. The present study suggests that combinational treatment of essential oil and radiation could be a novel promising approach for improving the shelf-life and safety of food and feed matrices. As well, study accomplishes that usage of natural antifungal agents with the combination of food-processing techniques is an appropriate and innovative strategy to improve decontamination efficiency of food-processing techniques.

## Author contributions

NK, JK, CS, KK, and VM designed and interpreted data of the work. NK, JK, CS, VG, and VM have drafted the work. All authors have approved the final version to be published.

### Conflict of interest statement

The authors declare that the research was conducted in the absence of any commercial or financial relationships that could be construed as a potential conflict of interest.
