# Supplementary material for: Combinational Inhibitory Action of Hedychium spicatum L. Essential Oil and γ-Radiation on Growth Rate and Mycotoxins Content of Fusarium graminearum in Maize: Response Surface Methodology
Source: Front Microbiol. 2018 Jul 31;9:1511. doi: 10.3389/fmicb.2018.01511 (PMC6079234; doi:10.3389/fmicb.2018.01511)
Supplement: Supplementary file 1 [file Table_1.docx]

**Supplementary Table 1:** Experimental range, levels, mean, and standard deviation of independent variables used in RSM design.

| Factor | Name | Units | Type | Range | |  | Levels | | | | |  | Mean | Standard deviation |
| --- | --- | --- | --- | --- | --- | --- | --- | --- | --- | --- | --- | --- | --- | --- |
|  |  |  |  | Minimum | Maximum |  | -2 | -1 | 0 | 1 | 2 |  |  |  |
| A | HSEO | mg/g | numeric | 0 | 3.15 |  | 0 | 0.25 | 1.57 | 2.89 | 3.15 |  | 1.57 | 0.99 |
| B | Radiation | kGy | numeric | 0 | 6.0 |  | 0 | 0.47 | 3 | 5.52 | 6 |  | 3.00 | 1.90 |
